# The impact of spontaneous cough on pleural pressure changes during therapeutic thoracentesis

**DOI:** 10.1038/s41598-022-15480-4

**Published:** 2022-07-07

**Authors:** Anna M. Stecka, Elżbieta M. Grabczak, Marcin Michnikowski, Monika Zielińska-Krawczyk, Rafał Krenke, Tomasz Gólczewski

**Affiliations:** 1 Nalecz Institute of Biocybernetics and Biomedical Engineering, Polish Academy of Sciences, 02-109 Warsaw, Poland; 2 Department of Internal Medicine, Pulmonary Diseases and Allergy, Medical University of Warsaw, 02-097 Warsaw, Poland

**Keywords:** Physiology, Diseases, Signs and symptoms

## Abstract

Cough during therapeutic thoracentesis (TT) is considered an adverse effect. The study was aimed to evaluate the relationship between cough during TT and pleural pressure (Ppl) changes (∆P). Instantaneous Ppl was measured after withdrawal of predetermined volumes of pleural fluid. Fluid withdrawal (FW) and Ppl measurement (PplM) periods were analyzed separately using the two sample Kolmogorov–Smirnov test and the nonparametric skew to assess differences between ∆P distributions in periods with and without cough. The study involved 59 patients, median age 66 years, median withdrawn fluid volume 1800 mL (1330 ÷ 2400 mL). In total, 1265 cough episodes were recorded in 52 patients, in 24% of FW and 19% of PplM periods, respectively. Cough was associated with significant changes in ∆P distribution (*p* < 0.001), decreasing the left tail of ∆P distribution for FW periods (the skew =  − 0.033 vs. − 0.182) and increasing the right tail for PplM periods (the skew = 0.182 vs. 0.088). Although cough was more frequent in 46 patients with normal pleural elastance (*p* < 0.0001), it was associated with significantly higher ∆P in patients with elevated elastance (median Ppl increase 2.9 vs. 0.2 cmH_2_O, respectively). Cough during TT is associated with small but beneficial trend in Ppl changes, particularly in patients with elevated pleural elastance, and should not be considered solely as an adverse event.

## Introduction

Pleural effusion affects approximately 1.5 million patients per year in the United States with the annual number of thoracenteses reported between 127,000 and 173,000^1–3^. Although, in general, therapeutic thoracentesis (TT) is thought to be a safe procedure, the withdrawal of a large pleural fluid volume can be associated with some complications, including chest discomfort, pain, pneumothorax and re-expansion pulmonary edema; some of these side effects are at least partially related to pleural pressure (P_pl_) fall caused by fluid withdrawal^4–6^. In patients with normal pleuro-pulmonary mechanics, a gentle slope of the withdrawn pleural fluid volume−P_pl_ curve reflects the replacement of the fluid by the expanding lung. However, if lung expandability is limited by, for example, visceral pleural thickening, lung scars, fibrosis, or airway collapse, even a small amount of withdrawn pleural fluid may result in significant P_pl_ decline, which in turn may produce symptoms such as vague chest discomfort or cough^7^. Although significant chest discomfort is believed to be an indication for TT termination because it may suggest a potentially unsafe decline of P_pl_^5–8^, the significance of cough seems to be controversial. Jones et al. recorded cough in less than 1% of patients undergoing ultrasound guided TT and it was not related to post procedure pneumothorax^9^; however these authors referred to other studies showing a significantly higher cough incidence (9–24%) and stated that cough in the late phase of TT should be regarded as an indication to stop the procedure. On the other hand, cough may be associated with lung re-expansion and thus, if not accompanied by other symptoms, it should not be considered as an indication for TT termination^5,10^.

Pleural manometry is a key tool to study different aspects of pleural pathophysiology in patients with pleural effusion. Access to pleural manometers, whether water or electronic, enables P_pl_ monitoring during TT and provides new insight into processes occurring during pleural fluid withdrawal, including a better understanding of complications and symptoms reported by the patients^11^, although its routine use in clinical practice in daily life was recently put into question^12^. Some previous observations led to the intriguing conclusion that cough during TT may favorably impact P_pl_ allowing to avoid excessive P_pl_ decline^10^. Although that observation was based on three patients only, it suggested that cough during TT need not necessarily be considered as a predictor of forthcoming complications, but may also be viewed as a protective phenomenon during the procedure. In general, we hypothesized that: (a) cough is linked with subsequent P_pl_ increase or its less significant decrease, (b) cough can be related to increased recruitment rate of atelectatic lung regions. Verification of the first hypothesis was the main purpose of this study.

## Results

Data of 63 patients who underwent TT with pleural manometry between January 2015 and January 2019 were reviewed. Records of 3 patients were excluded due to a questionable reliability of P_pl_ measurements caused by the presence of fibrin membranes and loculations (n = 2) and low quality records (n = 1). One patient was excluded due to unclear and uncertain cough markings in the study documentation. Thus, the records of 59 patients were included in the final analysis. Patients' characteristics are presented in Table 1.

**Table 1:** Characteristics of the study population.

Number of patients	59
Age; median (Q1 ÷ Q3) [years]	66 (58 ÷ 79)
Females; n (%)	35 (59)
Side of the pleural effusion, right/left; n/n	31/28
**Volume of pleural fluid in chest radiograph; n (%)** 1/3–2/3 of hemithorax More than 2/3 of hemithorax The entire hemithorax	20 (34) 24 (41) 15 (25)
The volume of withdrawn pleural fluid; median (Q1 ÷ Q3) [L]	1.8 (1.33 ÷ 2.4)
**Distribution of withdrawn pleural fluid volume; n (%)** ≤ 1500 mL 1501–2000 mL 2001–3000 mL 3001–4000 mL > 4000 mL	21 (36) 17 (29) 13 (22) 3 (5) 5 (8)
Malignant fluid etiology; n (%)	55 (93%)
Pleural elastance ≥ 14.5 cm H_2_O; n (%)	13 (22%)
Patients with cough during TT	52 (88%)

Cough during TT occurred in 52 patients. The total number of cough episodes was 1265 and the median and maximal number of episodes in individual patients were 11 and 104, respectively. Cough was found in 172 of 926 analyzed P_pl_ measurement periods (P_pl_M; 19%) and in 222 of 927 analyzed fluid withdrawal periods (FW; 24%). Characteristics of the changes in P_pl_ (∆P) distributions for P_pl_M and FW, with and without cough, are presented in Table 2.

**Table 2:** P_pl_ changes during measurement and fluid withdrawal periods with and without cough.

	P_pl_ measurement periods								
	Rare measurements phase (after every 200 mL of PF withdrawn)		P	Frequent measurements phase (after every 100 mL of PF withdrawn)		P	All measurements		P
Cough during P_pl_M (number of periods)									
Parameters of ΔP distribution	YES (n = 41)	NO (n = 245)		YES (n = 131)	NO (n = 509)		YES (n = 172)	NO (n = 754)	
Median ∆P [cm H_2_O]	0.3	0.0	< 0.05	0.3	0.2	< 0.001	0.3	0.1	< 0.001
Q1; Q3 of ∆P [cm H_2_O]	− 0.3; 0.9	− 0.3; 0.4		− 0.4; 1.5	− 0.3; 0.6		− 0.4; 1.4	− 0.3; 0.6	
Kurtosis	23	55		2	9		17	27	
Nonparametric skew	0.192	0.121		0.18	0.03		0.182	0.088	

	Fluid withdrawal periods								
	Rare measurements phase (every 200 mL of PF withdrawn)		P	Frequent measurements phase (every 100 mL of PF withdrawn)		P	All measurements		P
Cough during FW (number of periods)									
Parameters of ΔP distribution	YES (n = 44)	NO (n = 243)		YES (n = 178)	NO (n = 462)		YES (n = 222)	NO (n = 705)	
Median ∆P [cm H_2_O]	− 1.7	− 1.5	NS	− 0.5	− 0.9	< 0.001	− 0.7	− 1.1	< 0.001
Q1; Q3 of ∆P [cm H2O]	− 2.3; − 0.3	− 2.5; − 0.8		− 1.6; 0.8	− 1.8; − 0.1		− 1.8; 0.5	− 2.0; − 0.3	
Kurtosis	8	30		2	106		7	73	
Nonparametric skew	− 0.093	− 0.231		0.009	− 0.166		− 0.033	− 0.182	

PF—pleural fluid; n—number of periods with/without cough; ∆P—the difference between the end-expiratory P_pl_ values at the end and beginning of a period; Q1 and Q3—the first and third quartile, respectively; The two sample Kolmogorov–Smirnov test was used to assess statistical significance of differences between the ΔP distributions in periods with and without cough.

**Figure 1:**
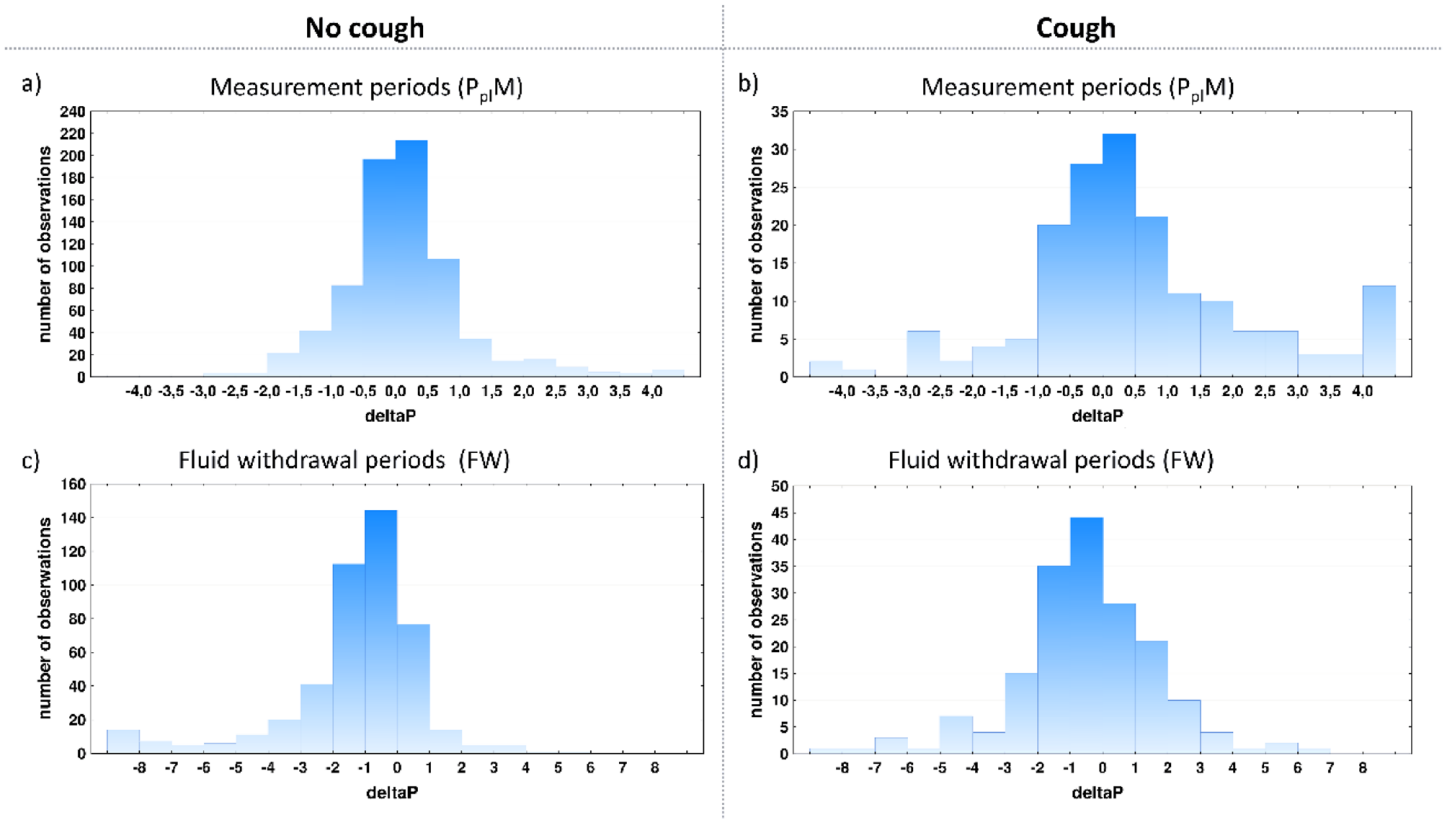
Influence of cough on the ∆P distribution during P_pl_ measurement periods (P_pl_M) (**a** and **b**) and during fluid withdrawal periods (FW) (**c** and **d**). (**a**) the distribution for P_pl_M without cough, (**b**) the distribution for P_pl_M with cough episodes, (**c**) the distribution for FW with no cough, (**d**) the distribution for FW with cough episodes. The figure was created using graph tool included in Statistica 13.1 software package (StatSoft Inc., Tulsa, USA) for which the required license has been purchased by the authors' institution.

There was a relationship between cough and ∆P both during P_pl_M and FW. Histograms of ∆P during P_pl_M showed that cough was associated with an increase of the right tail of the ∆P distribution (Fig. 1). This is quantitatively illustrated by nonparametric skew values, e.g., the skew for all P_pl_M without cough was equal to 0.088 vs. 0.182 for periods with cough (Table 2). During FW, cough was associated with a decrease of the left tail and an increase of the right tail of the ∆P distribution, resulting in an increase of the skew from − 0.182 for all periods without cough to − 0.033 for all periods with cough. Of note, the changes in the ∆P distribution were statistically significant only in the second phase of TT, i.e. the frequent measurement phase (Table 2).

Forty six and thirteen patients were found to have normal and elevated P_el_, respectively. There was a statistically significant difference in the number of cough episodes between these groups (*p* < 0.0001; Mann–Whitney U-test). The median number of coughs in normal and elevated P_el_ group was 22 and 1, respectively; ΔP was greater in patients with increased P_el_, particularly during P_pl_M with cough (Table 3). In both groups, the differences in the ∆P distributions between P_pl_M with and without cough were statistically significant (Table 3).

**Table 3:** The effect of cough during measurement periods on the ΔP distributions in patients with normal and elevated pleural elastance.

	Patients with normal pleural elastance (n = 46)			Patients with elevated pleural elastance (n = 13)		
Cough during P_pl_M (number of periods)						
Parameters of ΔP distribution	YES (n = 167)	NO (n = 660)	P	YES (n = 5)	NO (n = 94)	P
Median ∆P [cm H_2_O]	0.3	0.1	< 0.001	3.2	0.3	< 0.025
Q1; Q3 of ∆P [cm H_2_O]	− 0.4; 1.3	− 0.4; 0.5		2.9; 6.0	− 0.1; 1.1	

n—number of patients; ∆P—the difference between the end-expiratory P_pl_ values at the end and beginning of a period; Q1 and Q3—the first and third quartile, respectively; Two sample Kolmogorov–Smirnov test was used to assess statistical significance of differences between ΔP distributions in periods with and without cough.

## Discussion

Our study showed the association between cough and the pattern of P_pl_ changes during pleural fluid withdrawal. This is reflected by a significantly different ΔP distribution for periods with cough episodes compared to those with no cough. Cough was associated with the shift of ΔP towards more positive values during P_pl_M and less negative values during FW (Table 2, Fig. 1). Thus, the occurrence of cough seems to be associated with a beneficial trend in P_pl_ changes, making P_pl_ slightly higher or less negative than in periods without cough. An additional important observation is that cough during TT was significantly more common in patients with normal pleural elastance compared to those with elevated P_el._ Interestingly, albeit cough was far less common in patients with high P_el_, it resulted in significantly more pronounced increase in P_pl_ than in patients with normal P_el_ (Table 3). As, to our knowledge, this is the first study that provides reliable statistical data on the relationship between cough and P_pl_ changes during TT, we believe our paper adds to the existing literature on pleural physiology. In the context of our results, we can hypothesize that cough during TT can be perceived not only as an adverse effect of pleural fluid withdrawal but also as a factor contributing to a mechanism preventing the rapid and excessive decline of P_pl_.

Based on our previous observation^10^, two possible trends of cough-related P_pl_ changes during P_pl_M had been assumed: (1) an increase of P_pl_ due to lung re-expansion and (2) no significant changes if cough was not associated with additional lung re-expansion. It can be supposed that the withdrawal of the first several portions of pleural fluid in patients with large volume pleural effusion may not significantly reduce lung compression. Therefore cough in the initial phase of pleural fluid withdrawal may not generate significant P_pl_ changes until the space for lung re-expansion appears. Then, the increase in P_pl_ may be observed if cough helps to open the compressed alveoli and fill them with air. The above hypothesis seems to be confirmed by our results. We found that cough-associated increase in P_pl_ was more pronounced in measurements performed after withdrawal of 1 L of pleural fluid (Table 2). We believe this may be related to a more significant decrease in hydrostatic pressure and smaller lung compression, which may facilitate lung re-expansion. As, in general, cough is associated with significant intrathoracic pressure variations up to 400 cm H_2_O during the compression phase^13^*,* we may anticipate that such a P_pl_ fluctuation may facilitate lung recruitment. Without these significant P_pl_ fluctuations the recruitment is hindered due to such factors as the surface tension and viscosity of liquid layer in the collapsed bronchioles, alveolar ducts and alveoli^14,15^. According to Mead et al., a fast opening of the atelectatic lung regions may require an airway pressure as high as 140 cm H_2_O^16^. Considering the above, we postulate a direct influence of cough on lung recruitment.

**Figure 2:**
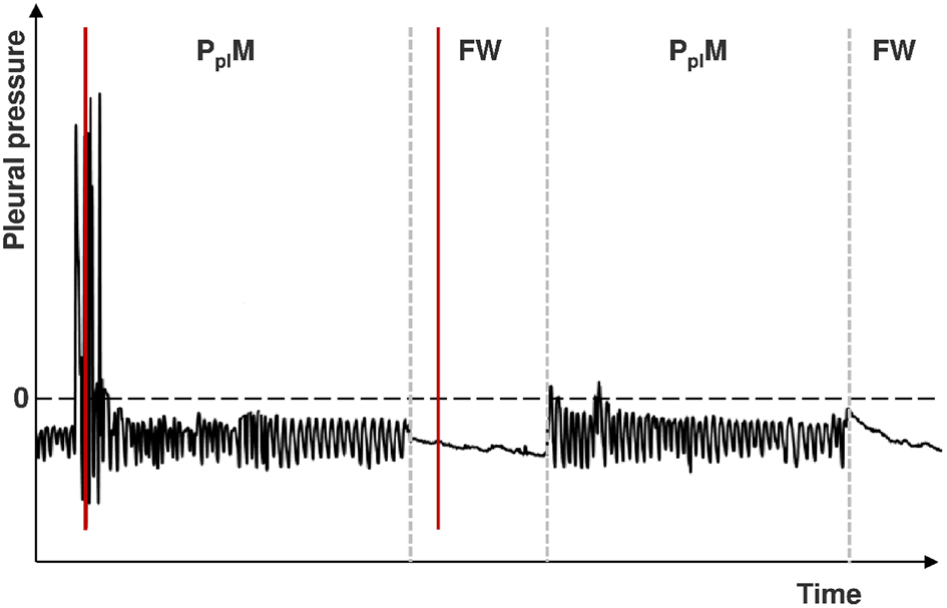
An example of pleural pressure record during therapeutic thoracentesis. The figure depicts 2 measurement periods (P_pl_M) and 2 fluid withdrawal periods (FW) with red, vertical lines outlined during the procedure indicating cough episodes during P_pl_M and FW. These markings were used to identify cough episodes during off-line analysis. Note that during P_pl_M the cough episode (red line) corresponds with peaks of P_pl_; in contrast, as P_pl_ is not registered during FW, cough can be identified exclusively by the marking drawn during the procedure. As depicted, no cough episodes occurred during the second P_pl_M.

The above suggestion seems to be supported by a recent study that showed some beneficial influence of continuous positive airway pressure (CPAP) applied during TT^17^. CPAP primarily results in an increase of airway pressure, with only a secondary increase of P_pl_. This implies the transpulmonary pressure increase^18^ that favors lung re-expansion. The impact of cough is probably much more complex. This is because cough also generates extremely high airway pressure but its rise is invariably associated with rapid contraction of the expiratory muscles and increase in P_pl_. Thus, in general, there is no increase in transpulmonary pressure unless the deep inspiration preceding the contraction (Fig. 2) is also considered. However, as cough is a complex mechanical phenomenon and there are sophisticated mechanical interactions between the lung compressed by pleural fluid and the second lung, the distribution of airway pressure might probably be different than in normal lungs. Hence, we can speculate that regional differences of airway pressure distribution and the differences in the dynamics of its changes in relation to P_pl_ may favor re-expansion of some lung regions and result in the change of natural pattern of P_pl_ decline. It should be admitted that the opposite relationship cannot be excluded, i.e. that lung re-expansion is responsible for cough generation. In other words, cough does not facilitate lung recruitment and is beneficial per se but it only reflects some factors or phenomena associated with lung re-expansion. In FW, P_pl_ declines due to fluid evacuation, and, therefore, ∆P has negative values. If, however, effects of atelectatic regions recruitment are comparable to hydrostatic pressure decrease caused by fluid withdrawal, ∆P has less negative or even positive values (Fig. 1c). As, according to our hypothesis, cough episodes facilitate lung expansion, the number of positive ∆P values increases and the number of negative ∆P values decreases for FW with coughs (Fig. 1d), and, in consequence, the value of non-parametric skew is less negative (Table 2).

Although, according to the literature^9^, cough is a symptom presented by 9–24% of patients undergoing TT (while 88% in our study), its mechanism remains unclear. It has been documented that the main receptors responsible for cough are present in the larynx, trachea and main bronchi^19^. The vagal afferents are also present in small bronchi and lung parenchyma (juxtapulmonary receptors), and the new ERS chronic cough guidelines mention potential existence of cough receptors also in the alveolar septa and parenchyma of the lungs^20^. However, there is no proof that their irritation results in cough, despite the fact that cough is one of the symptoms in patients with heart failure, pulmonary edema or altitude sickness. It is supposed that cough in those cases appears only when sputum moves to larger bronchi and irritates the cough receptors or when there is bronchial compression^21^. The presence of cough receptors in the pleura is also considered^4^. Some authors suggested that negative P_pl_ caused by fluid withdrawal stimulates cough receptors on the visceral pleura, particularly in patients with nonexpendable lung ^4,22^. The results of our study do not seem to support these opinions since cough was significantly less common in patients with elevated P_el_. Importantly, although less common, cough in patients with high P_el_ resulted in higher increase in P_pl_ than in patients with normal P_el_ (Table 3). Perhaps, lower (more negative) P_pl_ in those patients together with cough resulted in more effective and rapid recruitment of atelectatic lung regions, what may be supported by higher ∆P values observed also in P_pl_M without cough (Table 3). Thus, considering the above, we suggest that patients with elevated P_el_ may be further sub-classified into the two following groups: (a) sustainably elevated P_el_, and (b) elevated P_el_ which can be overcome by additional maneuvers e.g. cough or CPAP.

Some authors believe that cough is a criterion for TT termination as it is considered a sign of complete or near-complete drainage^23–25^. The results of our study do not support this opinion. This is because our study showed that in some patients a large fluid volume could have been withdrawn without significant P_pl_ fall despite episodes of cough which appeared even in the early phase of the procedure (data not published). Therefore, we agree with the opinion of Feller-Kopman et al. that TT should not be terminated solely because of cough^5^. Moreover, our findings may even support a hypothesis that cough can even be a beneficial factor preventing an excessive P_pl_ drop during TT in some cases. To confirm this view and to evaluate the clinical application of voluntary cough during thoracentesis, further, well-designed prospective studies are mandatory. It also seems necessary in the future to have a closer look to cough episodes characteristics in terms of P_pl_ increase; namely to check whether all types and episodes of cough can be construed as beneficial, especially cough attacks or severe excessive cough during the procedure.

**Figure 3:**
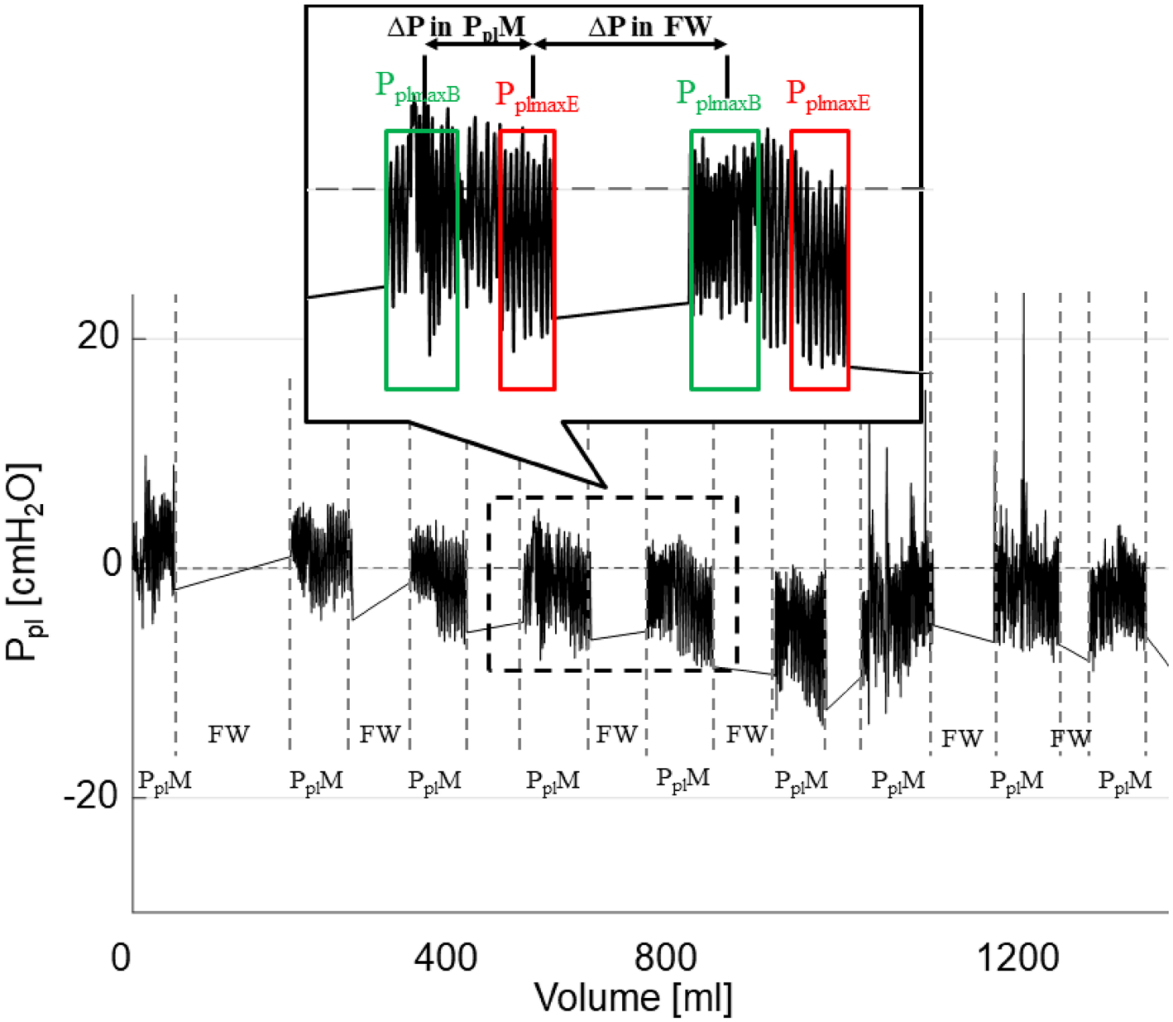
Graphic presentation of the method used to extract specific sections of the pleural pressure (P_pl_) curve for the assessment of P_plmax_ changes. P_pl_M- pleural pressure measurement period, FW–fluid withdrawal period, P_plmaxB_—maximal values of P_pl_ at the beginning of P_pl_ measurement period (the initial 2/5 of the entire P_pl_M), P_plmaxE_—maximal values of P_pl_ at the end of P_pl_ measurement period (the terminal 2/5 of the entire P_pl_M).

Our study has several limitations, with the most important being the retrospective nature of the analysis which can only prove statistical associations and does not allow us to assess other aspects of cough as e.g. the duration of its impact on Ppl changes. However, it should be stressed that the study hypothesis was formulated before the retrospective analysis had been performed and the data were evaluated in a predefined and well-designed manner. As previously mentioned, we could only calculate ΔP using the maximal value of P_pl_ for each breath as a surrogate of P_pl_ at FRC, whereas this value depends on the P_pl_ at FRC and possible intrinsic positive end-expiratory pressure (therefore, ΔP could be slightly negative in some P_pl_M–Fig. 3). Unfortunately, reliable estimation of the P_pl_ at FRC was impossible without continuous, simultaneous spirometry measurement to detect the expiration end. Also, the majority of the patients had malignant pleural effusion, as we focused on subjects requiring large therapeutic thoracentesis. Therefore, it was impossible to analyze if cough affects P_pl_ similarly in patients with benign pleural effusion. Finally, as the study was specifically focused on the relationship between cough and P_pl_, we have not analyzed other characteristics of cough during TT.

## Conclusions

Cough during TT is associated with small, beneficial trends in P_pl_ changes. This effect is particularly pronounced in patients with elevated P_el._ Although the true significance of cough-related increase in P_pl_ is difficult to estimate, the results of our study may suggest that cough during TT should not be considered solely as an adverse event. We believe that the effect of voluntary cough on the pattern of P_pl_ decline should also be tested in the context of its potential effect on elevation of P_pl_ and increase in the volume of pleural fluid that can be withdrawn.

## Methods

### General study design

This was a retrospective analysis of data collected from a cohort of patients who were enrolled in a larger, prospective project evaluating the impact of TT on cardiovascular and pulmonary function. This project was supported by the Polish National Science Centre (grant No 2012/05/B/NZ5/01343) and by the Nalecz Institute of Biocybernetics and Biomedical Engineering, Polish Academy of Sciences (the IBBE PAS own research fund). The study protocol was approved by the Institutional Review Board of Medical University of Warsaw (KB 105/2012) and registered at ClinicalTrials.gov (NCT02192138) on 16/07/2014. The study conformed to the standards set by the Declaration of Helsinki. All medical procedures were performed in patients hospitalized in the Department of Internal Medicine, Pulmonary Diseases and Allergy, Medical University of Warsaw, and all patients signed an informed consent to participate in the study.

### Patients

Sixty three patients who underwent TT with pleural manometry between January 2015 and January 2019 were included in the analysis; consecutive subjects were enrolled to avoid selection bias. The inclusion criteria were as follows: (1) age 18–85 years; (2) symptomatic pleural effusion occupying at least 1/3 of the hemithorax (in posteroanterior chest radiograph); (3) symptoms severity (dyspnea) warranting TT; (4) no contraindication for TT; (5) signed informed consent for participation in the study. The exclusion criteria were: (1) poor performance status requiring maximal shortening of the procedure; (2) unstable hemodynamic or respiratory status unrelated to pleural effusion; (3) respiratory failure requiring mechanical ventilation.

### Thoracentesis and pleural manometry

TT and pleural manometry were performed in sitting position, as described previously^26–29^. Pleural fluid was evacuated through a small-bore pleural catheter (Turkel™ Safety System, Covidien, Whiteley Fareham, UK). Pleural pressure was measured with a digital pleural manometer (IBBE PAS, Warsaw, Poland) and recorded for one minute directly after catheter insertion and then during the procedure. There were interchanging periods of P_pl_ measurement (P_pl_M) and periods of fluid withdrawal (FW) (Fig. 2). Initially, P_pl_ was measured after the withdrawal of each 200 mL up to 1L (the phase of rare P_pl_ measurements), and then after the withdrawal of each 100 mL (frequent P_pl_ measurement phase). The procedure was terminated when no more fluid could be aspirated, a significant pleural pressure decline was observed or chest pain occurred. P_pl_ was displayed on a monitor during the procedure and its instantaneous values were recorded on a portable computer for further analysis. Each cough episode, both during P_pl_M and FW, was marked in the computer record at the corresponding place on the time vector and noted in the patient's individual study documentation (Fig. 2).

### Data analyses

In each patient, all P_pl_M periods recorded during the procedure were analyzed to assess a possible influence of cough on the respiratory system. As instantaneous P_pl_ values depend on several variables, including respiratory muscle activity, the end-expiratory P_pl_ values (at functional residual capacity (FRC)) should ideally be used for analysis. This is because at FRC (i.e. after spontaneous, slow expiration) respiratory muscles are fully relaxed and P_pl_ depends entirely on the volume of pleural fluid and the relationship between the outward pull of the thoracic cavity and the inward elastic recoil of the lung. Since, however, the exact time points of expiration ends were impossible to determine without airflow measurement, the maximal value of P_pl_ (P_plmax_) in a breathing cycle was assumed to reflect P_pl_ at FRC. The median values of P_plmax_ at the beginning (P_plmaxB_) and the end (P_plmaxE_) of P_pl_M were used to quantitatively characterize P_pl_ changes during P_pl_M and FW. Thus, ∆P equal to the difference between P_plmaxE_ and P_plmaxB_ (P_plmaxE_–P_plmaxB_) was considered as the index characterizing the P_pl_ change during the corresponding P_pl_M, whereas ∆P equal to the difference between P_plmaxB_ for the subsequent P_pl_M and P_plmaxE_ for the previous P_pl_M was used to quantify the P_pl_ change during FW (Fig. 3). The first and last 2/5 of the P_pl_M period were treated as the beginning and as the end of the P_pl_M, respectively. This 2/5 was a compromise between the following requirements: (a) the number of P_plmax_ values used in determination of the median should be as large as possible to avoid random errors, and thus the interval for analysis should be relatively long; (b) P_plmaxB_ and P_plmaxE_ should reflect the P_plmax_ values at the actual beginning and end of each P_pl_M, and thus the intervals should be short and limited to extremes of the P_pl_M.

Additionally, to examine whether a possible link between cough and ΔP depends on lung expandability, all patients were classified according to the total pleural elastance (P_el_), i.e. the ratio of P_pl_ fall during the whole procedure to the total volume of withdrawn fluid. The first group included patients with normal P_el_ (< 14.5cm H_2_O/L)^30^, while the second included those with elevated elastance (≥ 14.5cm H_2_O/L).

### Statistical analysis

Statistical analysis was performed using the Statistica 13.1 software package (StatSoft Inc., Tulsa, USA). Data were presented as median and quartiles. Since the majority of analyzed variables had non-normal distributions, non-parametric statistical tests were used. The differences between groups were tested with the Mann–Whitney U-test. To analyze the statistical significance of cough influence on the ΔP distribution shape, the two-sample Kolgomorow-Smirnow test was used. The nonparametric skew was used to determine the nature of this influence. *P* values < 0.05 were considered statistically significant.

## Data Availability

The datasets used and analysed during the current study are available from the corresponding author on reasonable request.
