# Author Correction: The impact of spontaneous cough on pleural pressure changes during therapeutic thoracentesis

**DOI:** 10.1038/s41598-023-41308-w

**Published:** 2023-08-28

**Authors:** Anna M. Stecka, Elżbieta M. Grabczak, Marcin Michnikowski, Monika Zielińska-Krawczyk, Rafał Krenke, Tomasz Gólczewski

**Affiliations:** 1 Nalecz Institute of Biocybernetics and Biomedical Engineering, Polish Academy of Sciences, 02-109 Warsaw, Poland; 2 Department of Internal Medicine, Pulmonary Diseases and Allergy, Medical University of Warsaw, 02-097 Warsaw, Poland

Correction to: *Scientific Reports* 10.1038/s41598-022-15480-4, published online 07 July 2022

The Acknowledgements section in the original version of this Article was incomplete.

“The authors thank Piotr Korczyński, PhD MD, Krzysztof Zieliński, PhD Eng. and Krzysztof J Pałko, PhD Eng., for their assistance during the clinical phase of the study. We would like to thank Marta Maskey-Warzęchowska for her editorial assistance.”

now reads:

“The authors thank Piotr Korczyński, PhD MD, Krzysztof Zieliński, PhD Eng. and Krzysztof J Pałko, PhD Eng., for their assistance during the clinical phase of the study. We would like to thank Marta Maskey-Warzęchowska for her editorial assistance. The study was partially supported by funds from the National Science Centre, Poland (Grant No. 2019/35/B/NZ5/02531).”

The original Article has been corrected.

